# Is p38 MAPK Associated to Drugs of Abuse-Induced Abnormal Behaviors?

**DOI:** 10.3390/ijms21144833

**Published:** 2020-07-08

**Authors:** Rana El Rawas, Inês M. Amaral, Alex Hofer

**Affiliations:** Experimental Addiction Research, Department of Psychiatry, Psychotherapy and Psychosomatics, Division of Psychiatry I, Medical University Innsbruck, 6020 Innsbruck, Austria; ines.amaral@i-med.ac.at (I.M.A.); a.hofer@i-med.ac.at (A.H.)

**Keywords:** p38 MAPK, cocaine, conditioned place preference, reward, stress, anxiety, depression, nucleus accumbens, social interaction, k opioid receptors

## Abstract

The family members of the mitogen-activated protein kinases (MAPK) mediate a wide variety of cellular behaviors in response to extracellular stimuli. p38 MAPKs are key signaling molecules in cellular responses to external stresses and regulation of pro-inflammatory cytokines. Some studies have suggested that p38 MAPK in the region of the nucleus accumbens is involved in abnormal behavioral responses induced by drugs of abuse. In this review, we discuss the role of the p38 MAPK in the rewarding effects of drugs of abuse. We also summarize the implication of p38 MAPK in stress, anxiety, and depression. We opine that p38 MAPK activation is more closely associated to stress-induced aversive responses rather than drug effects per se, in particular cocaine. p38 MAPK is only involved in cocaine reward, predominantly when promoted by stress. Downstream substrates of p38 that may contribute to the p38 MAPK associated-behavioral responses are proposed. Finally, we suggest p38 MAPK inhibitors as possible therapeutic interventions against stress-related disorders by potentially increasing resilience against stress and addiction relapse induced by adverse experiences.

## 1. Introduction

**Figure 1:**
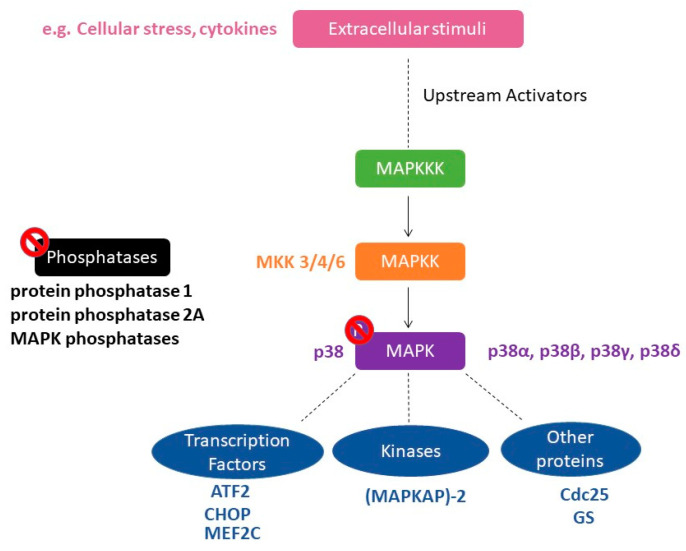
Schematic representation of the p38 mitogen-activated protein kinases (MAPK) signaling pathway. A variety of extracellular signals, such as cellular stress and cytokines, can activate the p38 MAPK pathway. This leads to the initiation of MAPK phosphorylation cascade, in which MAPKKK phosphorylate the p38 MAPK-specific MAPKKs MKK3, MKK4, or MKK6. Subsequently, the four isoforms of p38 MAPK (α, β, δ, and γ) are phosphorylated, thereby activating various p38 MAPK substrates. Substrates can be transcription factors such as ATF2, CHOP, and MEF2C, protein kinases such as (MAPKAP)-2 as well as other proteins such as Cdc25 and GS. Phosphorylated substrates go on to elicit varied biological responses. The duration of signaling is controlled by phosphatases such as protein phosphatase 1; protein phosphatase 2A or MAPK phosphatase. Abbreviations: CHOP, CCAAT/enhancer-binding protein-homologous protein; MEF2C, myocyte enhancer factor 2C; (MAPKAP)-2, MAPK-activated protein; GS, Glycogen synthase.

The mitogen-activated protein kinases (MAPK) superfamily is made up of three majorsignaling pathways: The extracellular signal-regulated protein kinases (ERKs), the c-Jun N-terminal kinases or stress-activated protein kinases (JNK/SAPK), and the p38 family of kinases. The MAPK is a serine/threonine kinase that is activated through phosphorylation by a MAPK kinase (MKK), which is a “dual-specific” kinase that phosphorylates at both serine/threonine and tyrosine residues within a threonine/any amino acid/tyrosine (Thr X Tyr) motif, in which the middle amino acid is different for each MAPK [1]. Enzymes in the p38 MAPK module are subject to dual phosphorylation at the Thr-Gly-Tyr motif situated within the kinase activation loop and are primarily activated by various environmental stresses, including heat, osmotic and oxidative stresses, as well as inflammatory cytokines [2] (Figure 1).

p38 MAPK is inactive in the non-phosphorylated state. Dual phosphorylation at Thr-180 and Tyr-182 residues by either MKK3 or MKK6 induces global conformational reorganizations that modify the alignment of the C- and N-terminal domains of p38 MAPK, consequently permitting the binding of ATP and the desired substrate [3,4,5]. However, selective activation by distinct MKKs has been observed among p38 isoforms, as evidenced by the inability of MKK3 to effectively activate one isoform of p38, p38β, while MKK6 is a potent activator [5,6]. MKK4 can also activate p38 in vitro or when co-expressed with p38 in cultured cells, although reported as a non-physiologic activator of p38 [2]. Phosphorylated p38 MAPK (pp38) can activate a wide range of substrates. These include transcription factors such as ATF2, CCAAT/enhancer-binding protein-homologous protein (CHOP), and myocyte enhancer factor 2C (MEF2C), as well as protein kinases such as the MAPK-activated protein (MAPKAP)-2, and cytosolic and nuclear proteins such as Cdc25 and Glycogen synthase (GS) [2,5]. Duration of signaling is controlled by phosphatases, including protein phosphatase 1, protein phosphatase 2A, or MAPK phosphatases [5]. Phosphorylated substrates go on to elicit varied biological responses that include inflammation, apoptosis, proliferation, cell-cycle regulation, and differentiation [5,7]. The growing list of specific downstream targets and various activation modes of the p38 MAPK pathway raise a possibility of the functional diversity of each p38 MAPK isoform [7]. Four different genes encoding p38 MAPK isoforms (p38α, p38β, p38γ, p38δ) with > 60% overall sequence homology and > 90% identity within the kinase domains have been described in human tissues [2]. Of these, p38α and p38β are ubiquitously expressed while p38γ and p38δ are differentially expressed depending on tissue type [6]. Generally, p38α and p38β are highly expressed in the brain with p38α being mainly expressed in neuronal cells and p38β being highly expressed in both neuronal and glial cells [7]. At the subcellular level, p38α is distributed in dendrites, in cytoplasmic and nuclear regions of the cell body of neurons, in contrast to p38β that is preferentially expressed in the nucleus of neurons [7]. 

The pyridinylimidazole compounds, represented by SB203580, were initially seen as inflammatory cytokine synthesis inhibitors [8,9]. Subsequently, they were found to be selective inhibitors of p38 MAPK [8,9]. SB203580 inhibits the catalytic activity of p38 MAPK by competitive binding at the ATP binding site of the kinase [8,10]. MAPK inhibitors appear to be a therapeutic strategy in several disease models, particularly inflammatory disorders, due to their capability to reduce the synthesis and the signaling of pro-inflammatory cytokines [11]. This review discusses the role of p38 MAPK in the rewarding effects of drugs of abuse, as well as in stress-related behaviors.

## 2. Role of p38 MAPK in the Rewarding Effects of Drugs of Abuse

**Figure 2:**
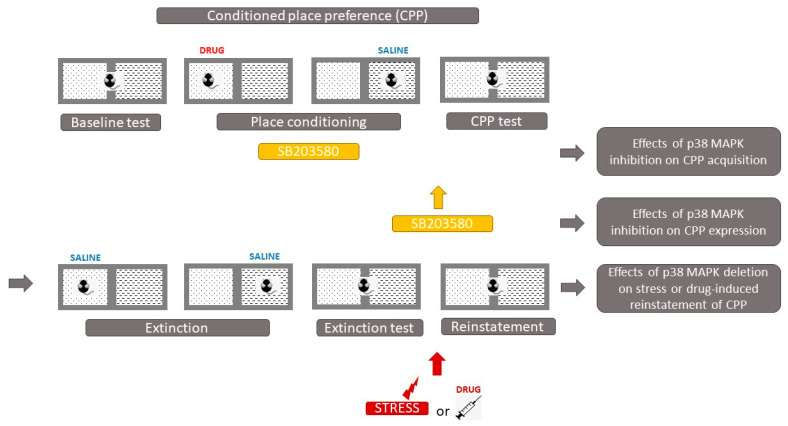
conditioned place preference (CPP). In the pre-conditioning day or baseline test, animals (such as mice) are allowed to freely explore the entire apparatus. The time that mice spend in each compartment is recorded to show the natural preference of animals. During conditioning, mice are randomly assigned to their drug and saline training compartments for the training sessions. Conditioned preference is assessed by allowing the mice to roam freely in all the compartments and recording the time spent in each. When the p38 MAPK inhibitor, SB203580, is injected before DRUG conditioning (the stimulus), the effects of p38 inhibition on CPP acquisition are investigated. Likewise, when SB203580 is injected before the CPP test, the effects of p38 inhibition on CPP expression are explored. After the CPP expression, mice can undergo extinction of CPP, during which they are conditioned repeatedly to saline in both compartments. By conditioning the animals with saline in their previous DRUG-associated compartment, mice progressively lose their preference to the compartment associated to the DRUG. When the animals reach the extinction criteria, which is checked by an extinction test, they can be tested for reinstatement of CPP. Reinstatement can be induced by an exposure to stress (stress-induced reinstatement of CPP) or also, by an exposure to a priming injection of the DRUG (drug-induced reinstatement). Place preference during reinstatement of CPP is again determined by allowing the mice to freely explore all the compartments of CPP.

Environmental stimuli associated with drugs of abuse motivate animals to “prefer” the contexts associated with drug intake and to spend more time in these contexts. Such an approach, known as conditioned place preference (CPP), is widely used for evaluating the rewarding effects of drugs [12,13]. During CPP, the animal is “conditioned” in an experimental apparatus consisting of at least two compartments that have distinct visual and tactile cues. The animal can be injected with the drug in one compartment and with saline in the other compartment. The neutral environment associated with the drug acquires secondary motivational properties, such that it can act as a conditioned stimulus when the animal is subsequently exposed to this environment again [14]. During the test, the animal can “choose” the compartment in which it prefers to spend more time. If the drug has rewarding properties, the animal will “prefer” to spend more time in the compartment previously associated with the drug (Figure 2). 

The nucleus accumbens (NAc) is a critical element of the mesocorticolimbic system which has a well-established role in mediating the rewarding effects of drugs of abuse [15]. Gerdjikov et al. tested the hypothesis that the inhibition of MAPKs would inhibit the acquisition of NAc amphetamine CPP. They showed that NAc injections of the ERK inhibitor PD98059 or the p38 kinase inhibitor SB203580 dose-dependently impaired CPP, but not the JNK inhibitor SP600125 [16]. These results suggest that ERK and p38, but not JNK, MAPKs may be necessary for the establishment of NAc amphetamine-produced CPP [16]. Moreover, Zhang et al. showed that repeated morphine treatment induced the acquisition of CPP and increased the phosphorylation of p38 in the NAc [17]. Consistently, the microinjection of the p38 inhibitor SB203580 into the NAc prior to the administration of morphine prevented the acquisition of CPP and inhibited the activation of p38, thereby indicating that the activation of p38 in the NAc may be necessary for morphine CPP [17]. The same group also found that, following 5 days of morphine treatment, p38 activation was induced in the NAc microglia but not in astrocytes or neurons [18]. They reported that the bilateral microinjection of minocycline, a putative inhibitor of microglia, or SB203580, a selective p38 MAPK inhibitor, into the NAc before each morphine treatment for five days impaired the acquisition of morphine CPP and suppressed the activation of p38 signaling in the microglia induced by morphine treatment [18]. Following the acquisition of morphine CPP, a single minocycline or SB203580 injection failed to block the expression of morphine CPP [18]. Thus, based on these studies, it appears that p38 signaling in the NAc microglia may play an important role in the acquisition but not the expression of morphine CPP [18]. Therefore, it has been proposed that p38 MAPK in the NAc is involved in abnormal behavioral responses induced by drugs of abuse [17]. Later, another study also showed that intraperitoneal (i.p.) injections of SB203580 dampened the acquisition of morphine-induced CPP in mice and reduced the phosphorylation of p38 MAPK in the NAc of morphine CPP mice [19]. 

The effect of p38 MAPK inhibition on cocaine-induced behaviors, such as the acquisition and expression of CPP, was also investigated. The expression of cocaine CPP was specifically blocked by i.p. SB203580 when injected on the post-conditioning test day [20]. By contrast, morphine CPP was not affected by i.p. SB203580 [20]. Thus, in line with previous studies, i.p. inhibition of p38 failed to block the expression of CPP to morphine. On the other hand, studies that performed intracerebroventricular (icv) injections of SB203580 before cocaine training found no effect on cocaine CPP [21,22]. Consistently, the deletion of p38α in the serotonergic neurons of the dorsal raphe nucleus (DRN) [23] or the conditional knock-out of p38α MAPK in the dopaminergic neurons of the ventral tegmental area (VTA) [24] does not affect cocaine CPP, suggesting that the deletion of p38α does not alter the associative learning required for place preference or the rewarding properties of cocaine. Interestingly, serotonergic p38α MAPK deletion blocked the reinstatement of cocaine preference induced by stress, but not by cocaine priming, thereby showing that serotonergic p38α MAPK deletion selectively alters only the stress-induced modulation of cocaine-seeking behaviors [23]. Table 1 summarizes the studies investigating p38 MAPK blockade on drug reward. 

**Figure 3:**
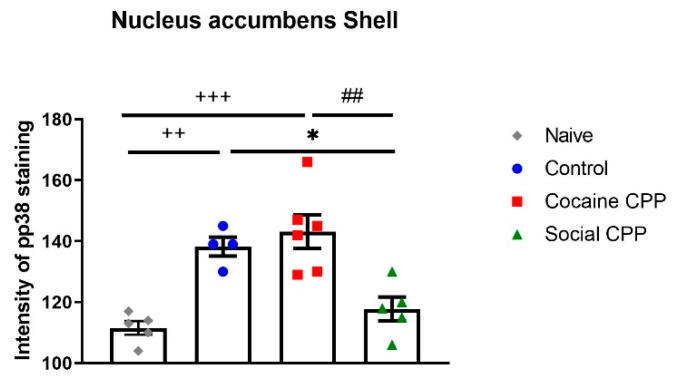
pp38 expression in the NAc shell 24 h after the cocaine CPP test [34]. Social interaction CPP decrease p38 MAPK activation to the levels of naïve untreated rats. Control rats receiving saline in both compartment of CPP and cocaine CPP expressing rats show similar increased levels of pp38 as compared to naïve untreated and social CPP expressing rats. Statistical test: one-way analysis of variance followed by Tukey’s post hoc test. * *p* < 0.05, different from saline control; ++ *p* < 0.01, +++ *p*< 0.001 different from naïve; ## *p*< 0.01 different from cocaine CPP.

Similarly to drugs of abuse, rats acquire and express CPP for natural reward such as social interaction [25,26,27,28,29,30,31]. In general, social reward CPP is assessed by placing the rats during half the conditioning sessions into a compartment of the CPP with their assigned social partner, and during the other half of the sessions alone into the other compartment of the CPP [26,27,28,32]. We and others [33] perform social interaction reward CPP by pairing one compartment with a sex- and weight-matched male conspecific preceded by an i.p. injection of saline for 15 min and the other compartment with saline only [32]. Animals that spent more time in the social interaction-paired compartment than in the saline-paired compartment during the CPP test expressed preference for social interaction. When comparing social interaction CPP to cocaine CPP, almost the same brain regions were activated [31]. However, parts of the insular cortex, namely the granular insular cortex and the dorsal part of the agranular insular cortex, were more activated after cocaine CPP, whereas the prelimbic cortex and the core sub-region of the NAc were more activated after social interaction CPP [31]. Given that p38 MAPK activation was reported to be increased after morphine CPP, we assessed the expression of p38 MAPK activation in the NAc shell and core sub-regions at different time points after cocaine CPP and compared it to social interaction CPP [34]. It was expected that cocaine CPP would enhance the activation of p38 in the NAc as compared to social interaction CPP and to control rats that received saline in both compartments of the CPP. However, we found that control rats and cocaine CPP expressing rats showed similarly enhanced p38 activation compared to naïve untreated and social interaction CPP rats. Furthermore, 24 h after social interaction CPP, pp38 neuronal levels in the NAc shell decreased to the level of naïve untreated rats with pp38 expressed mainly in neurons (92%) (Figure 3).

**Figure 4:**
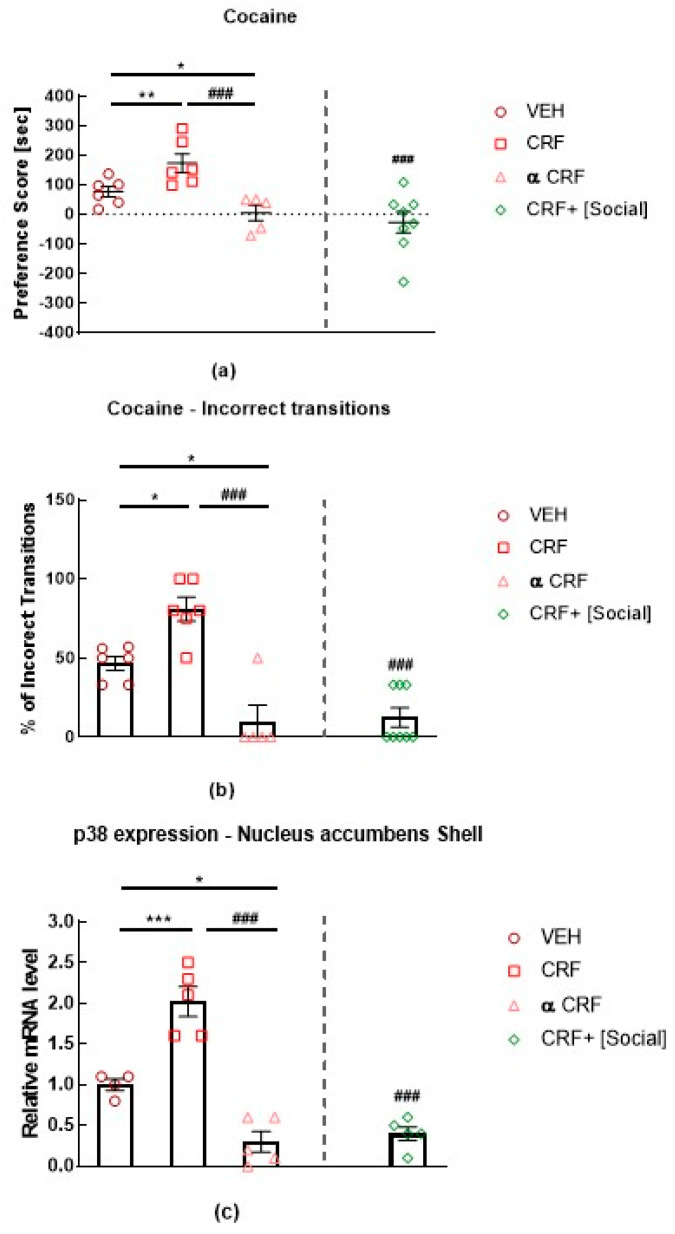
Effects of intracerebroventricular (icv) injections of vehicle, corticotropin-releasing factor (CRF), and alpha-helical CRF (α CRF) on (**a**) cocaine preference; on (**b**) associated percentage of incorrect transitions and on (**c**) associated p38 expression in the NAc shell [22]. Preference score is the time that the rat spent in the stimulus-associated compartment during the test-pretest. CRF+ [social] is a group of rats conditioned with cocaine that received icv injections of CRF prior to cocaine conditioning but also had the opportunity to social interaction in the alternative compartment of the CPP. The incorrect transitions of cephalocaudal grooming progression were evaluated during the test session of CPP for each treatment condition. Statistical test: one-way analysis of variance followed by Tukey’s post hoc test. α CRF, alpha-helical CRF; VEH, vehicle * *p* < 0.05, ** *p* < 0.01, *** *p* < 0.001 different from VEH; ### *p* < 0.001, different from CRF.

As p38 plays a role in stress and anxiety behaviors that will be detailed in the coming section, these results suggest that: First, cocaine treatment per se does not induce p38 activation as control rats and cocaine CPP-expressing rats show the same levels of pp38 in the NAc shell. Second, marginal stress such as injecting animals with saline and placing them into the CPP apparatus is sufficient to induce p38 activation in the NAc shell. In fact, compared to naïve untreated animals, control rats receiving saline injections in both compartments of the CPP, expressed significantly higher levels of activated p38 in the NAc shell. Finally, social interaction reward has anti-stress effects as social interaction-expressing CPP rats show levels of pp38 similarly to naïve untreated rats [34]. Indeed, rats receiving corticotropin-releasing factor (CRF) icv injections before cocaine conditioning showed an increase in cocaine CPP, whereas those receiving icv injections of the non-selective antagonist alpha-helical CRF (α CRF) showed a decrease in cocaine CPP [22]. Remarkably, when social interaction was made available in the alternative compartment, CRF-induced increase of cocaine preference was reversed completely to the level of rats receiving cocaine paired with α CRF. This reversal of cocaine preference was also paralleled by a reversal in altered behavioral sequencing of grooming, considered as a marker of stress [35] and by a CRF-induced increase of p38 MAPK expression in the NAc shell [22] (Figure 4). These results show that the modulation of the CRF system has a direct impact on p38 MAPK expression in the NAc shell, as p38 MAPK expression was increased after icv CRF injections prior to each cocaine conditioning and was decreased after icv injections of α CRF prior to each cocaine conditioning [22]- Figure 4. Accordingly, p38 is considered to be more closely related to stress modulation than to cocaine treatment. Indeed, CRF was reported to induce dynorphin-dependent k opioid receptor (KOR) activation in the NAc [36] with p38 being an important mediator of the KOR-dependent aversive properties of stress [21,37].

We propose that the anti-stress effects of social interaction might be mediated by the KOR system in the ventral NAc shell, which was previously shown to drive aversion via KOR activation [38] through a decrease in the activation of p38 MAPK [34]. In parallel, we additionally propose that the rewarding effects of social interaction might be mediated by the KOR system in the dorsal NAc shell, which has also previously been shown to drive preference/reward via KOR activation [38] through an increase in ERK.

## 3. Role of p38 MAPK in Stress, Anxiety, and Depression

Sustained stressful experience can lead to maladaptive responses, including clinical depression, anxiety, and an increased risk for drug addiction [39,40]. After stress exposure, p38 MAPK is generally activated in different brain regions (Table 2). Repeated forced swim stress activated p38 MAPK in the cortex, the hippocampus, and the NAc [21], which decreased significantly following a pretreatment with SB203580 before bouts of forced swimming [21]. pp38 levels were also increased in the prefrontal cortex (PFC) after cold exposure [41], in the hippocampus after enhanced single prolonged stress [42] and in the DRN after social defeat stress [23]. Additionally, a significant positive correlation was found between early life stress and the percentage of monocytes staining positive for pp38 [43]. Neuro-inflammation, in response to bacterial endotoxin lipopolysaccharide (LPS)-induced depressive-like behaviors, has been reported to be accompanied by increased levels of pp38 in the habenula [44]. Both the p38 inhibitor SB203580 and the anti-depressant fluoxetine normalized the changes in p38 phosphorylation and reversed the depressive-like behaviors [44]. Interestingly, the depletion of neuronal p38α in mice resulted specifically in increased anxiety-related behaviors without affecting learning and memory processes or motor coordination and muscle function [45].

p38 activation appears to be an important mediator of KOR-induced aversive stress effects through G-protein-coupled receptor kinase 3 (GRK3)/β-arrestin, a KOR-associated protein, dependent mechanisms [21,37]. Inhibition of p38 MAPK was found to block stress-induced behavioral responses, including aversive responses to the KOR agonist U50, 488 [21,46]. Importantly, cell-specific deletion of p38α MAPK in serotonergic neurons blocked stress-induced aversion [23]. These effects seem to be regulated by KOR as KOR knockout (KO) mice did not develop conditioned place aversion (CPA) to U50,488; however, re-expression of KOR in the serotonergic neurons of the DRN or in the dopaminergic neurons of the VTA of KOR KO mice activated p38 and restored place aversion [24,47].

**Figure 5:**
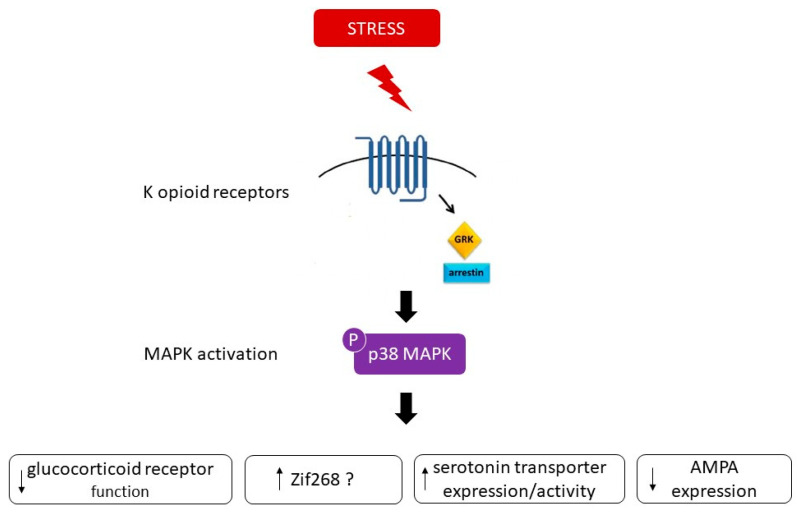
Arrestin-dependent signaling events result in p38 MAPK activation and subsequent dysphoric behavioral responses. Possible substrates encoding the behavior responses to stress downstream to p38 comprises changes in gene expression such as zif268, in serotonin transporter expression/activity, in glucocorticoid receptor function and modulation of proteins related to synaptic plasticity, such as AMPA receptors.

One possible mechanism encoding the behavior responses to stress is a change in gene expression downstream to p38 [21]. One candidate is zif268, whose induction is p38-dependent [21] and has previously shown to be a direct downstream target of p38 [48]. Indeed, multiple swim stress exposure has been reported to cause a significant up-regulation of zif268 in the striatum only in wild type but not in KOR^(−/−)^ mice [21]. SB203580 had an inhibitory effect on zif268 induced by stress, suggesting that this immediate early gene may be involved in the aversive responses to KOR activation [21]. Another possible substrate of pp38 that may contribute to the behavioral responses is the serotonin transporter (SERT). In fact, p38 MAPK activation has been reported to regulate the activity of SERT in vitro [49,50]. Evidence of an in vivo relationship between activation of p38 MAPK signaling pathways and central serotonin function/metabolism was described by [43]. They found a significant negative correlation between the percentage of monocytes staining positive for intracellular pp38 and CSF concentrations of the serotonin metabolite 5-HIAA in non-human primates [43]. Thus, the activation of the p38 pathway would be expected to decrease synaptic availability of serotonin and to reduce the serotonin metabolites by increasing SERT expression/activity [43]. Further evidence was provided in mice demonstrating that SERT activity in nerve terminals of serotonergic neurons is positively modulated in a p38α dependent manner [23]. Stress-induced p38α MAPK caused translocation of SERT to the plasma membrane in the brain, thereby increasing the rate of transmitter uptake at serotonergic nerve terminals and inducing a hypo-serotonergic state that underlies depression-like and drug-seeking behaviors [23]. In line with these findings, cytokine induction by LPS produced SERT activation and behavioral despair, both requiring p38 MAPK pathway activation [51]. In mice exhibiting a selective elimination of p38α MAPK in serotonergic neurons, LPS failed to elevate brain SERT activity despite normal peripheral stress responses [52]. Moreover, p38α MAPK excision in serotonergic neurons resulted in behavioral resilience to anxiety and depression-like behaviors [52]. Thus, p38 activation can stimulate the expression of SERT, which is used as a major pharmacological target for depression treatment [44]. A third possible candidate could involve the modulation of proteins related to synaptic plasticity, such as AMPA receptors. p38 MAPK signaling has been shown to be an important mediator of AMPA receptor surface trafficking during synaptic plasticity in which activation of p38 MAPK may lead to synaptic removal of surface AMPA receptors [53,54]. Generally, compounds which augment signaling through AMPA receptors exhibit antidepressant-like behavioral effects in animal models [55]. For example, the antidepressant fluoxetine has been found to alter AMPA receptor phosphorylation in a manner that is expected to increase AMPA receptor signaling [55]. Therefore, it is possible that a decrease in the surface expression of AMPA receptors contributes to the behavioral responses associated to p38 MAPK. One additional target of p38 relevant to depression could involve the glucocorticoid receptor (GR). It was reported that GR function is reduced in patients with major depression [56]. p38 signaling pathways have been shown to be implicated in the inhibition of GR function. Indeed, activation of p38 MAPK has been demonstrated to disrupt transactivation of the GR [57], leading potentially to glucocorticoid resistance or decreased responsiveness to glucocorticoids, a primary feature of major depression [58]. Therefore, SB203580 through inhibition of p38 MAPK could recover the normal functioning of GR and alleviate the glucocorticoid resistance underlying depression [44]. Figure 5 summarizes the possible substrates that may contribute to the behavioral responses associated to p38 MAPK. 

## 4. Conclusions

It is unclear why p38 MAPK is merely involved in the expression of cocaine CPP when the inhibitor is administered before the post-conditioning test in a drug-free state [20], i.e., when the animals encounter drug-associated cues, but not in the learning required for the rewarding properties of cocaine. It was previously observed that KOR activation before the presentation of cocaine-associated cues enhances approach behaviors to those cues [59], possibly via activation of p38 signaling pathway. This potentiation of cocaine CPP by KOR activation does not result from an enhancement of associative learning mechanisms, as KOR activation only occurred before the final preference test after the associative learning phases were already complete. Conversely, the inhibition of p38 signaling only after the post-conditioning test might reduce the rewarding value of cocaine-associated contexts. More studies are needed to emphasize this possibility, in particular because expression of cocaine CPP did not increase the levels of pp38 in the regions of NAc core or NAc shell [34]. Yet, the study by [20] suggested that p38 MAPK-mediated norepinephrine transporter (NET) up-regulation is linked to cocaine-induced CPP. 

It appears that p38 MAPK activation is more closely associated to stress-induced aversive responses rather than drug effects per se. Mostly, studies show that p38 MAPK activation is only involved in cocaine reward, predominantly when promoted by stress. However, it remains open to discussion how p38 MAPK is implicated in CPP morphine acquisition. The first explanation could be that morphine might activate KOR as well as µ opioid receptors (MOR). Indeed, it has been reported that morphine is weakly selective to the MOR and possesses affinity to δ opioid receptors (DOR) and KORs [60,61]. This explanation is further supported by the fact that naloxone, a non-selective opioid antagonist, could block the acquisition of morphine CPP [62]. However, the rewarding effects of morphine are abolished in MOR-deficient animals [63], thereby showing that MOR gene product is the molecular target of morphine in vivo. In addition, the k-opioid antagonist nor-binaltorphimine did not affect morphine CPP [64]. Remarkably, it appears that DORs, rather than KORs, are implicated in the acquisition of morphine reward; as the administration of the selective delta-2-opioid receptor antagonist naltriben prior to morphine was able to block morphine-induced CPP [65], suggesting that this first explanation is unlikely to occur. The second explanation might be that opioid receptor-mediated p38 phosphorylation has also been demonstrated for MORs [66]. MOR opioids could to some extent induce activation of p38 [67,68]. It is therefore plausible that inhibition of p38 signaling during morphine training could abolish acquisition but not before the post-conditioning test, after that morphine acquisition was already established.

In conclusion, understanding the molecular and cellular mechanisms that control stress-induced behaviors could explain the neurobiological mechanisms involved in depression and addiction-like behaviors and provides insight to potential therapeutic targets. Emerging evidence demonstrates a role for p38 MAPK in depression, anxiety, and addiction relapse induced by stress. Targeting the p38 MAPK pathway for therapeutic advantage might appear standard, given the broad range of pathologies in which this pathway is implicated. However, the pathology-specific functions and targets of p38 MAPK together with its interaction with other intracellular regulatory pathways initiates many challenges to exploiting this pathway for therapeutic benefit [5]. Indeed, p38 MAPK inhibitors have been studied extensively in both preclinical experiments and clinical trials for inflammatory diseases. Here, we opine that p38 MAPK inhibitors are of growing interest as possible therapeutic interventions against stress-related disorders by potentially increasing resilience against stress and addiction relapse induced by adverse experiences.

## Figures and Tables

**Table 1:** Summary of the findings describing the role of p38 MAPK in drug reward.

Drug	P38 Blockade	Results	References
Amphetamine	SB203580 into the NAc	impaired amphetamine conditioned place preference (CPP)	[16]
Morphine	SB203580 into the NAc	prevented the acquisition but not the expression of morphine CPP	[17,18]
Morphine	SB203580 i.p.	dampened the acquisition of morphine-induced CPP	[19]
Morphine	SB203580 i.p.	failed to block the expression of morphine CPP	[20]
Cocaine	SB203580 i.p.	blocked the expression of cocaine CPP	[20]
Cocaine	SB203580 icv	did not affect acquisition to cocaine CPP	[21,22]
Cocaine	deletion of p38α MAPK in the serotonergic neurons	did not affect cocaine CPP	[23]
Cocaine	conditional knock-out of p38α MAPK in the dopaminergic neurons	did not affect cocaine CPP	[24]
Cocaine	serotonergic p38α MAPK deletion	blocked the reinstatement of cocaine preference induced by stress	[23]
Cocaine	serotonergic p38α MAPK deletion	did not affect the reinstatement of cocaine preference induced by cocaine priming	[23]

**Table 2:** Summary of the findings reporting the activation p38 MAPK after CPP to drugs and exposure to stress.

Stimuli/Protocol	Regions	Animals	References
morphine CPP	NAc	rats	[17,18]
		mice	[19]
repeated forced swim stress	cortex, hippocampus, NAc	mice	[21]
cold exposure	PFC	rats	[41]
enhanced single prolonged stress	hippocampus	rats	[42]
social defeat stress	dorsal raphe nucleus (DRN)	mice	[23]
early life stress	monocytes staining for pp38	monkeys	[43]
bacterial endotoxin LPS	habenula	rats	[44]

## Abbreviations

ERK	Extracellular signal-regulated protein kinases
JNK	c-Jun N-terminal kinases
MAPK	Mitogen-activated protein kinases
MKK	MAP kinase kinase
NET	Norepinephrine transporter
pp38	Phosphorylated p38
CHOP	CCAAT/enhancer-binding protein-homologous protein
MAPKAP	MAPK-activated protein
MEF2C	Myocyte enhancer factor 2C
GS	Glycogen synthase
GRK3	G-protein-coupled receptor kinase 3
CPP	Conditioned place preference
KO	Knock out
NAc	Nucleus accumbens
i.p.	Intraperitoneal
icv	Intracerebroventricular
DRN	Dorsal raphe nucleus
VTA	Ventral tegmental area
CRF	Corticotropin-releasing factor
KOR	κ opioid receptor
αCRF	Alpha helical CRF
VEH	Vehicle
PFC	Prefrontal cortex
LPS	Lipopolysaccharide
CPA	Conditioned place aversion
SERT	Serotonin transporter
GR	Glucocorticoid receptor
MOR	µ opioid receptor
DOR	δ opioid receptor
